# Fragmented QRS formation in nonhypertensive acromegaly patients

**DOI:** 10.55730/1300-0144.5451

**Published:** 2021-12-09

**Authors:** Muhammet DURAL, Göknur YORULMAZ, Kadir Uğur MERT, Selda MURAT

**Affiliations:** 1 Department of Cardiology, Eskişehir Osmangazi University Faculty of Medicine, Eskişehir, Turkey; 2 Department of Endocrinology, Eskişehir Osmangazi University Faculty of Medicine, Eskişehir, Turkey

**Keywords:** Fragmented QRS, electrocardiography, acromegaly

**To the Editor**,

We would like to indicate our satisfaction with your interest in our article [1]. It has been shown that fQRS formation is associated with poor clinical outcomes in many diseases, especially in coronary artery disease [2,3]. The presence of fQRS is an indicator of myocardial fibrosis, so myocardial dysfunction development is more common in patients with fQRS on electrocardiography (ECG) and the cardiovascular prognosis is worse [4,5].

Studies have shown that the extent and localization of fQRS on ECG are among the factors that show the extent of myocardial damage [6,7]. Since acromegaly is a rare disease and our study was a single-center study, the number of patients in our study was relatively small. Another factor in the small number of patients was that the acromegaly patients with hypertension were not included. Since there were not enough number of patients, we could not make a separate analysis to evaluate the relationship between fQRS extent and localization and left ventricular hypertrophy (LVH) parameters. Of course, if the number of our patients was sufficient and we could do these analyzes, the value of our study would increase.

In our study, we showed that LVH parameters in acromegaly patients with fQRS (+) were higher than those with fQRS (-). These results show that patients with acromegaly who have fQRS on their ECG should be followed more closely for the development of acromegalic cardiomyopathy and cardiovascular disease.

## References

[1] Dural M Yorulmaz G Alagüney ES Mert KU Çamli E Assessment of fragmented QRS formation and its relationship with left ventricular hypertrophy in non-hypertensive acromegaly patients Turkish Journal of Medical Science 2021 51 5 2437 2444 10.3906/sag-2101-229 PMC8742487 33992041

[2] Dural M Demir L Babayigit E Junushova B Mert KU Fragmented QRS formation and its predictors in patients with breast cancer receiving anthracycline-based chemotherapy Journal of Electrocardiology 2019 54 5 9 10.1016/j.jelectrocard.2019.02.003 30782548

[3] Eyuboglu M Fragmented QRS as a Marker of Myocardial Fibrosis in Hypertension: a Systematic Review Current Hypertension Reports 2019 21 10 73 10.1007/s11906-019-0982-3 31451958

[4] Das MK Suradi H Maskoun W Michael MA Shen C Fragmented wide QRS on a 12-lead ECG: a sign of myocardial scar and poor prognosis Circulation: Arrhythmia and Electrophysiology 2008 1 4 258 268 10.1161/CIRCEP.107.763284 19808417

[5] Das MK Saha C El Masry H Peng J Dandamudi G Fragmented QRS on a 12-lead ECG: a predictor of mortality and cardiac events in patients with coronary artery disease Heart Rhythm 2007 4 11 1385 1392 10.1016/j.hrthm.2007.06.024 17954396

[6] Eyuboglu M Kucuk U Senarslan O Akdeniz B Comparison of the presence of fragmented QRS complexes in the inferior versus the anterior leads for predicting coronary artery disease severity Revista Portuguesa de Cardiologia 2017 36 2 89 93 10.1016/j.repc.2016.07.008 28153633

[7] Torigoe K Tamura A Kawano Y Shinozaki K Kotoku M The number of leads with fragmented QRS is independently associated with cardiac death or hospitalization for heart failure in patients with prior myocardial infarction Journal of Cardiology 2012 59 1 36 41 10.1016/j.jjcc.2011.09.003 22019275

